# Gynecological and Obstetrical Society of Baltimore

**Published:** 1894-07

**Authors:** William S. Gardner

**Affiliations:** Secretary; 613 Park Avenue


### GYNECOLOGICAL AND OBSTETRICAL SOCIETY OF BALTIMORE.

### SIXTY-SECOND REGULAR MEETING.

*The President, Dr. Thos. A. Ashby, in the Chair.*

DR. GEORGE A. ROHE read the following paper upon "Hematoma of the Ovary."

Abdominal surgeons not infrequently find in extirpated ovaries small blood-clots, varying in size from a pea to a hazel-nut. The nature of these clots seems not very clearly understood. In most cases they are believed to be due to excessive hemorrhage into the Graafian follicle after rupture, and the escape of the ovule. This view seems to me not tenable, because in not a few instances no rupture of the follicle has occurred. Besides, the corpus luteum, the successor of the ovule in the occupancy of the Graafian follicle, frequently contains no blood. Indeed, the view seems not irrational that hematoma of the ovary, no matter how small it may be, should always be regarded as a pathological formation, having no essential connection with the physiological process of ovulation. In such a specimen as that here shown, in which the blood-clot in the fresh state of the specimen was as large as a small chestnut, we have to deal with a pathological condition. The specimen is from a case of hystero-epilepsy of over eight years duration, in which both ovaries and tubes were removed by abdominal section in 1891. The patient recovered, and has had no recurrence of the epileptic attacks for over two years. Ovaries presenting this appearance are not rarely seen in abdominal section. I am informed that some surgeons simply extirpate the hematoma, stitch up the wound in the ovary, and drop the organ back into the pelvis. I may be permitted to express doubt whether any good purpose is served by this so-called "conservative" surgery. In all cases of this kind that have come under my notice, there were either adhesions or displacements of the ovaries which are among the recognized indications for removal of these organs. Dr. B. F. Baer, who is known as a very careful and conservative surgeon, says in reference to these cases:* "Diseased ovaries, when due to hemorrhage into the Graafian follicles to such an extent as to produce the condition known as ovarian hematoma, should be removed. They cause intense suffering, and there is no other means of relief."

Dr. Mary A. Dixon Jones, of Brooklyn, and Dr. Francis Ferster are of the opinion that hematoma of the ovary is preceded by conditions termed by them "gyroma" and "endothelioma." Indeed, the latter writer, basing his opinion upon somewhat extended microscopical study of ovaries, normal and pathological, claims that "what previously was called a corpus luteum is invariably an endothelioma." That the corpus luteum is an endothelial structure may be accepted without dispute; that it should be called by a name heretofore applied to a malignant new formation, or that the consequences attributed by Ferster to this body hitherto considered so innocent, really follow in many cases, is, I think, open to grave doubt. Chronic oöphoritis and peri-oöphoritis, endarteritis and sclerosis are mentioned as histological findings, and pain and distress as clinical manifestations due to ovaries undergoing these morbid changes.

Dr. Ferster connects the corpora lutea with the production of hematomata as follows: "In my own experience a large number of so-called copora lutea of menstruation are endotheliomata of a pathological type. They grow under the influence of a chronic oöphoritis without coming to a typical end, or gradually increasing in bulk and frequently leading to the formation of hematoma under incessant local and constitutional trouble.

It will, I think, be generally conceded that a hemorrhage into an ovarian follicle, or into ovarian stroma, does not take place when the ovary or the blood-vessels preserve their normal structural integrity. Some nutritional change must have preceded the hemorrhage. It is most reasonable to believe that this change is in the blood-vessels of the ovary. Whether this nutritional disturbance is due to new formations properly dignified by the names "gyroma" and "endothelioma," or whether it is simply the result of chronic inflammation, is a question that must be referred to the pathologists for further investigation. Rollin,† who has recently

* Proceedings Philadelphia Obstetrical Society, June 12, 1892.

† Des Hemorrhagies de l'Ovaire, Paris, 1889—Frommel's Jahreabericht.

made a study of ovarian hematoma, gives chronic oöphoritis as a local condition antedating the hemorrhage.

While the occurrence of small collections of blood in the Graafian follicles, and minute extravasations in the ovarian stroma is not infrequent, the cases of so-called ovarian apoplexy, where the entire ovary is converted into a blood-cyst, varying from a billiard ball to a fetal head in size, are much more rare. The case presently to be related shows, however, that there is no essential difference between the two classes of cases.

The case referred to is as follows:

E. L., born in the United States, white, aged twenty-one years, single, was admitted to the Maryland Hospital for the Insane November 18, 1893. Until a month before admission there had been no mental disturbance beyond hysterical attacks of varying severity, sometimes accompanied by convulsions. Her disposition was usually amiable, although she was of rather unstable temper. Her habits were always industrious. So far as was ascertained there was no hereditary predisposition to insanity. The hysterical outbreaks were usually coincident with the menstrual periods, and have only been present for the past four or five years. Up to a year ago her physical condition was very good, but for three years she has suffered with a good deal of pain during the catamenia. About a year ago she consulted a gynecologist, under whose care she remained for several months with apparent improvement. During the last three or four weeks before admission a great change in her behavior was noticed. She became exalted, talkative, silly in conversation and action. When admitted she carried a large doll which she caressed and talked to in a childish manner. She was neat and cleanly in dress and habits and never noisy or maniacal. No apparent sexual excitement. At the end of two weeks she had lost all her delusions and was apparently restored to her normal mental condition. At the approach of the next menstrual period she became hysterical, had several convulsions, foamed at the mouth, screamed, or lay with eyes staring or closed. Reflexes normal. During these attacks she was unquestionably conscious of what was going on around her. One evening she set fire to her clothing, but the fire was promptly extinguished, and only a slight superficial reddening of small areas of the skin was produced. No serious results followed this attempt at self-destruction.

After the period was over, her normal mental condition returned, but she did not improve physically. She lost appetite, had nausea, and became thin and anemic.

The pains in the iliac region persisted and became especially severe on the left side. Occipital headache, rhachialgia and pain in the limbs, with attacks of nausea and vomiting were also present.

On January 18, 1894, a vaginal examination demonstrated an elastic swelling behind and to the left of the uterus, which was exquisitely sensitive to the touch. To the right there appeared to be an enlarged and prolapsed ovary. The uterus was adherent posteriorly, but somewhat movable.

The clinical diagnosis of adherent uterus, prolapsed ovary on the right and cystic ovary or ovarian abscess on the left side, was made, and an operation for the relief of these conditions recommended to her and her consent readily obtained. Inasmuch as she was, and had been for some weeks, entirely rational, her own consent was considered sufficient authority to proceed.

Abdominal section was done on January 28, 1894. Passing two fingers through the incision down to the fundus uteri this was found adherent, the tubes and ovaries on both sides being also bound down by adhesions. After carefully separating the latter, the right ovary, enlarged to the size of an English walnut, was brought up, ligated together with the thickened tube close to the uterus, and removed. In place of the left ovary was a cystic tumor as large as a mandarin orange, which ruptured as it was brought out of the abdominal wound and discharged a lot of softly-coagulated blood. My first thought was of an ectopic pregnancy, but as an examination of the specimen will show, this was a mistake and an unjust suspicion. After the tube and remains of the cyst were ligated and removed, the peritoneal cavity was flushed out with hot, distilled water and the abdominal wound closed with silkworm gut sutures. No drainage.

The subsequent course was uneventful, except that on the second day the temperature rose to 101° F., and the pulse to 102.

After a purgative enema of magnesium sulphate and glycerine, this slight disturbance vanished.

The stitches were removed on the seventh day, and the wound was found dry and thoroughly united. Patient out of bed on the twenty-first day.

Since the operation the patient has suffered no pain, is cheerful and industrious, not hysterical, and has gained flesh. Her mental condition apparently normal. The patient was discharged, entirely recovered, March 15, 1894.

The walls of the blood-cyst are apparently composed of ovarian stroma; the tube is somewhat thickened, but contains no pus. The right ovary, on section, shows two blood-clots about the size of hazel-nuts, apparently occupying unruptured Graafian follicles. This case seems to show on the two sides examples of two forms of ovarian hematoma which are, however, rarely associated in the same individual. If any conclusion can be drawn from a single case, it is that the rather common follicular hematoma and the infrequent ovarian apoplexy are identical in origin.

Winkel* refers to three cases of follicular hemorrhage into the ovaries after severe burns. The burn which my patient received about a month before the operation might be considered suggestive, if it had been more serious. The firm adhesions were, however, evidence of a longer duration, at least of the local inflammatory condition.

Of the more recent cases reported is one by Doran in Volume 32 of the Transactions of the London Obstetrical Society. Doran considered it a hemorrhage into the ovarian stroma from rupture of a follicle. The cyst-wall was one-eighth of an inch thick and consisted of ovarian stroma. Dr. Mundé† briefly reports a case of hematoma of both ovaries, one being the size of an orange and the other of a hen's egg. Dr. E. E. Montgomery,‡ in commenting on this case, refers to a similar one under his observation. Duncan§ reports a case in which there was hematosalpinx in connection with

* Frauen Krankheiten, 2 Aufl. p. 700.

† American Journal of Obstetrics, June, 1890, p. 638.

‡ Sajous Annual, 1891, II., G. 46.

§ Ibid., 1893, II., G. 5.

the ovarian hematoma. The history of the case suggests ectopic pregnancy, which seems, however, to have been excluded.

I am reminded here of a case which I saw about twelve years ago in the service of the late Dr. A. F. Erich at the Maryland Woman's Hospital. The patient was a white, single woman, thirty-five years of age. The tumor, supposed to be an ovarian cystoma, was about the size of a fetal head and when brought to the abdominal incision and tapped with the trocar, thick, black blood was evacuated. The patient died of purulent peritonitis about the fifth day, and at the autopsy a perforation of the rectum was found. How this was produced cannot be cleared up.

It may have been torn through in separating adhesions. A number of apparently similar cases, in which the cyst ruptured and caused death from septic peritonitis, are recorded by Bernutz and Goupil, but most of these were probably cases of extra-uterine pregnancy.

An ovarian hematoma may rupture and give rise to a pelvic hematocele. In other cases the bleeding may continue and the patient die of hemorrhage. The most serious danger from rupture is, however, peritonitis and sepsis. I am informed by Dr. Joseph Price that the contents of an ovarian hematoma are usually exceedingly virulent and liable to cause septic peritonitis if the blood-cyst is allowed to rupture within the peritoneal cavity.

The diagnosis of ovarian hematoma cannot be definitely made before abdominal section. Even when rupture occurs and a hematocele is formed, the diagnosis rests between several conditions, often differentiated with the greatest difficulty, even after operation. The only rationally indicated procedure is removal of the affected organ by abdominal section.

DR. ASHBY: I have had some specimens of marked hematoma of the ovary. Clinically these patients complain of much more pain than from other diseases. Most of these cases have been complicated with pelvic peritonitis. If the clinical history indicates violent dysmenorrhea the ovaries should be removed.

DR. NEALE asked if it be necessary to remove the organ for these small clots.

DR. WILLIAM S. GARDNER: I wish to notice briefly a few points concerning which Dr. Rohe makes statements that are at variance with what I believe.

In speaking of the pathology of hematoma, the essayist presumes that they are caused by diseased blood-vessels but admits that reliable pathologists have not settled this as a fact. Nevertheless, he proceeds as if it were a fact and advocates the complete removal of all ovaries that have even a small hematoma in them. In this he ignores some of the best work of the last six years. Martin alone has done twenty-seven operations in which, after completely removing one ovary, he resected the other ovary on account of small cysts or hematomæ. Twenty-four of these patients were completely cured without further treatment; they did not have to suffer the torments of the artificial menopause; they did not lose their sexual feeling; they were not rendered sterile; eight of the twenty-four bore children.

In another place Dr. Rohe makes the statement that hematomæ are frequently found associated with "adhesions or displacements of the ovaries which are among the recognized indications for removal of these organs." These statements are both true, but "recognized indications" are not necessarilly correct indications; if they were, all progress would stop. Polk has demonstrated beyond a doubt that these cases can be cured without the removal of the appendages. By Polk's method are these patients not only relieved of their pain, but they retain all their functions as women.

I do not wish to be understood as objecting to the removal of ovaries hopelessly disorganized either by hematoma or other process of disease, but it should be remembered that according to the testimony of Goodell, Glavaecke, Keith and many other authorities, the complete removal of the ovaries not only does *not* cure many cases of mental diseases, but that it actually causes a considerable number. Frequent as are these mental disturbances, they are among the minor evils resulting from the complete loss of both ovaries. The loss of sexual sense, the loss of the possibility of reproduction, and the multitude of discomforts of the artificial menopause, combine to make the life of many of these patients utterly miserable. I have upon my records the histories of patients operated upon by almost every operator in this city who have come to me with the complaints of their post-operative condition; and only too many of these patients have said that if they had known what they would have to suffer they would have preferred to keep both their ovaries and their pains. I make these statements only to remind those gentlemen who seem to forget it, that when a woman's ovaries are removed she is not yet freed from all the ills that may burden her existence.

---

SIXTY-FIFTH MEETING.

*The President, Dr. Thos. A. Ashby, in the Chair.*

DR. JAMES M. CRAIGHILL read a paper entitled "The Effect of Bicycle Riding on the Female Pelvic Organs."

The very extensive use of the bicycle by both sexes and the increasing number of female riders naturally cause the medical advisers to inquire into this popular sport and consider as to the advisability of its indulgence, or, like the sewing-machine, should it be condemned?

Much has been written in the daily press for and against this form of sport, also some little in the various medical journals of this country and Europe, but I have been unable to find a word relative to the female cyclist after reference to all the medical writings at my disposal. During the past six or seven years observations among my own patients, and acquiring as much information as I could collect about other female cyclists, leads me to think that the exercise is very beneficial to them, especially with the improved machines of the present day, and should be encouraged in moderation.

It is needless to say that this form of athletic sport, like any other, can be very much abused; such as overtaxing the muscles by trying to ride hills too steep, riding too far and too fast, not sitting properly on the wheel, and many other ways that might be mentioned. The fad among the male riders at the present time is to have the handle-bars of the machine so low that many of them sit with their bodies at an angle of forty-five degrees. While it is obvious this is a very injurious custom in many ways for the male, it would be much worse for the female, and as far as I know none of the latter sex have been so foolish as to adopt that position.

Nothing will more rapidly improve that class of anemic women which every medical man meets in his daily rounds suffering from backache, ovarian pains, leucorrhea, etc., caused by a general relaxed condition of the pelvic organs, in unison with a general run-down condition, than proper exercise, and it is the custom among physicians to advise women suffering in this way to exercise in the open air, this being regarded as much more likely to do good than the various tonics that are prescribed in such cases. The exercise usually consists of a walk of possibly a mile for the first day or two, but becoming tiresome is not tried long enough to do good; if our patient is put on a bicycle she soon becomes very enthusiastic, and indulges whenever an opportunity presents itself, and if properly instructed, wearing loose clothing, with no corsets, sitting erect on her wheel, exercising every organ and muscle in her body, as her course naturally leads her out of the city, she gets the benefit of the pure country air, and her exertions make her breathe in much larger quantities than ordinarily, thus purifying her blood and adding health and strength to that part of her body to which our attention is directed in this paper. This is a very different picture from her sister bending over the sewing-machine, usually in a close room, with her corset drawn tightly, crowding all of the abdominal contents down on her pelvic organs, with the subsequent congestion and the many female troubles with which we are all familiar.

Horseback riding is probably the next best exercise to the bicycle, but from a financial as well as gymnastic point of view, also general convenience, the cycle is the best.

Of course there are many conditions of the female organs that would prohibit this exercise, which are unnecessary to mention, but any condition that would admit of the equestrian exercise would be much benefited, and in most troubles in which any kind of self-exertion would be of benefit can, for the reasons given, be safely prescribed.

It is even customary with some to ride during the menstrual period and apparently with no harm resulting, although the writer of this paper would include riding at that time among the abuses.

A few cases of pregnant women riding have come under my care, and while the number of cases are too small to arrive at any definite conclusions, still it is the writer's opinion, if the woman has been accustomed to the exercise before she became in that condition, it will not injure her to continue it with proper care during the first six months of her gestation. A novice would run great risk of doing herself much injury in that condition from the exertion, numerous falls, etc., due to inexperience.

One of my patients, a well developed young woman, had been riding her wheel several years before her marriage and continued to do so after she had become pregnant and until she was about six months advanced, notwithstanding she had been cautioned by me to desist. Her wheel (at that time the best to be had) was of the solid tire pattern, heavy and hard to propel, and using no care in straining when riding up hills or over rough roads, she had a right occipito-posterior position. After a difficult labor, I delivered her with forceps with a resultant badly torn perineum and bowel, which was repaired by a secondary operation. After her recovery she again took to the wheel and is one of the best female cyclists in this city to-day, and has never had the least uterine trouble since that date now five years ago, although she has had several abortions which the writer has reason to think were brought on intentionally on her part.

Another patient had been riding a number of years before marriage and continued the exercise regularly up to two months before the birth of her child. She rode a very easy-running wheel, with pneumatic tire, and during the last few months was on a tandem wheel with her husband.

My former experience with a pregnant bicyclist caused many misgivings on my part about her riding at all after she became in that condition. She continued to do so after being warned not to pull up hills or exert herself very much at any time. The instructions were obeyed and her confinement was in every respect normal, with very little pain, and one of the easiest labors I ever attended. There has been no subsequent uterine trouble.

The history of the next case was gotten from the husband, as she has never been treated by me.

Mrs. L., aged twenty-seven, mother of two children, had suffered much from uterine trouble, probably a partial procidentia, with great pain during menstrual period. Before commencing the use of the wheel she had been treated by several physicians for her trouble and had found some relief from a pessary.

She had ridden very little before the birth of her first child and had an extremely difficult labor. When pregnant with her second child, rode up to the fifth month and at her second delivery had just the reverse of her first, an exceedingly easy time of it. During the past seven years has exercised regularly on her wheel and has had no uterine trouble whatever. While the writer admits this woman may have been cured by becoming a mother, still I am inclined to attribute much of her improved health to the outdoor exercise on her wheel.

I could mention other cases where the patients suffered much from dysmenorrhea until they adopted the wheel for exercise and then suffered very much less during their periods or were free from pain entirely.

While the few cases I have cited in this brief paper prove very little, still I thought in writing it I might call the attention of the members of this Society to the many good effects to be derived from this very attractive sport and health-giving exercise.

WILLIAM S. GARDNER,
613 *Park Avenue.* Secretary.

---

PATHOLOGY OF SCARLET FEVER.—Berge (*Union Med.*) considers scarlet fever a local infection due to streptococcus. These organisms are cultivated in the crypts of the tonsils, and there secrete a toxin, the diffusion of which throughout the organism produces the cutaneous and mucous eruptions. Puerperal and traumatic scarlet fever result from local infection of the uterine surface, or various other mucous or cutaneous surfaces, by the streptococcus. These conclusions were based on the following facts: The scarlet fever eruption follows the affection of the tonsils; the existence of scarlet fever with eruption in which the tonsillitis and its specific complications are the only affections; the constancy of the streptococcus in the tonsillitis of scarlet fever; the streptococcal nature of the complications of scarlet fever; the relation of scarlet fever to puerperal infection; and, lastly, the ease with which the erythema-producing properties of the streptococcus can be demonstrated.—*Ont. Med. Jour. Ex.*

